# Isolated cardiac muscle contracting against a real-time model of systemic and pulmonary cardiovascular loads

**DOI:** 10.1152/ajpheart.00272.2023

**Published:** 2023-09-15

**Authors:** Amy S. Garrett, Jarrah Dowrick, Andrew J. Taberner, June-Chiew Han

**Affiliations:** ^1^Auckland Bioengineering Institute, The University of Auckland, Auckland, New Zealand; ^2^Department of Engineering Science and Biomedical Engineering, The University of Auckland, Auckland, New Zealand

**Keywords:** cardiovascular, diastole, energetics, trabeculae, work-loop

## Abstract

Isolated cardiac tissues allow a direct assessment of cardiac muscle function and enable precise control of experimental loading conditions. However, current experimental methods do not expose isolated tissues to the same contraction pattern and cardiovascular loads naturally experienced by the heart. In this study, we implement a computational model of systemic-pulmonary impedance that is solved in real time and imposed on contracting isolated rat muscle tissues. This systemic-pulmonary model represents the cardiovascular system as a lumped-parameter, closed-loop circuit. The tissues performed force-length work-loop contractions where the model output informed both the shortening and restretch phases of each work-loop. We compared the muscle mechanics and energetics associated with work-loops driven by the systemic-pulmonary model with that of a model-based loading method that only accounts for shortening. We obtained results that show simultaneous changes of afterload and preload or end-diastolic length of the muscle, as compared with the static, user-defined preload as in the conventional loading method. This feature allows assessment of muscle work output, heat output, and efficiency of contraction as functions of end-diastolic length. The results reveal the behavior of cardiac muscle as a pump source to achieve load-dependent work and efficiency outputs over a wider range of loads. This study offers potential applications of the model to investigate cardiac muscle response to hemodynamic coupling between systemic and pulmonary circulations in an in vitro setting.

**NEW & NOTEWORTHY** We present the use of a “closed-loop” model of systemic and pulmonary circulations to apply, for the first time, real-time model-calculated preload and afterload to isolated cardiac muscle preparations. This method extends current experimental protocols where only afterload has been considered. The extension to include preload provides the opportunity to investigate ventricular muscle response to hemodynamic coupling and as a pump source across a wider range of cardiovascular loads.

## INTRODUCTION

The process of isolating muscles from the ventricles for experimental purposes decouples them from the in vivo hemodynamics of the body. Experimentalists thus attempt to replicate physiological hemodynamic loads by imposing contraction and shortening patterns ex vivo. In such experiments, isolated muscle preparations are typically mounted with one end attached to a length controller and the other to a force transducer. The length controller manipulates muscle length throughout each contraction, generally informed by feedback from the force transducer. It is common practice to design force-length controls that allow the muscle to generate force-length work-loops. Examples of these protocols include constant afterload (1–4), sinusoidal (2, 5, 6), and model-driven time-varying afterload (7–9) approaches. Such force-length work-loop protocols are designed to recouple isolated cardiac muscle preparations with the mechanical impedance they experience in vivo.

However, these current approaches consider only the ventricular ejection mechanics dictated by ventricular outflow hemodynamics and make little attempt to simulate the inflow hemodynamics associated with venous return. Thus, only the shortening phase of the work-loop is replicated, an approach that does not fully emulate in vivo conditions. In vivo, left ventricular (LV) muscles are impeded by the “afterload” of the systemic circulation and “preloaded” by the mechanics of the right ventricle and pulmonary circulation. The systemic impedance affects ventricular ejection and, hence, shortening of LV muscles, whereas the pulmonary system modulates ventricular refilling and, hence, restretching of LV muscles. In existing force-length work-loop protocols, contributions from ventricular inflow hemodynamics are ignored and, instead, isolated muscle preparations are restretched arbitrarily to a prescribed end-diastolic length. Changes in ventricular output affect distribution of blood throughout the system, impacting ventricular refilling. An experimental work-loop loading protocol that can capture the hemodynamics of the complete systemic-pulmonary system is thus required to study isolated LV muscles in vitro.

This study presents a method to recouple ex vivo LV trabeculae with the hemodynamics of the complete systemic-pulmonary system using a real-time model. We developed a real-time computational model that consists of a venous, arterial, and pump compartment for each side of the circulatory system. The pressure in the active pump compartments (ventricles) was calculated using the force generated by an isolated trabecula in vitro, and the pressure in the remaining venous and arterial compartments was computed from their time-varying volume and elastance. Blood flow between compartments resulted from the pressure gradients and resistances to flow. The volume change of the left ventricular compartment predicted by the model was then converted, via Laplace’s law, to a length change that was immediately, in real time, imposed on the trabecula.

As a case study, we compared the mechanoenergetics of three example muscles observed during work-loops with a “fixed end-diastolic length” protocol, contracting against a three-element Windkessel model of only systemic afterload, with those observed when the muscle experienced systemic-pulmonary loads defined by our model. This novel experimental approach enables, for the first time, the examination of the hemodynamic coupling between arteries and ventricular musculature in an in vitro setting.

## METHODS

### Constructing the Systemic-Pulmonary Model

**Figure 1.**
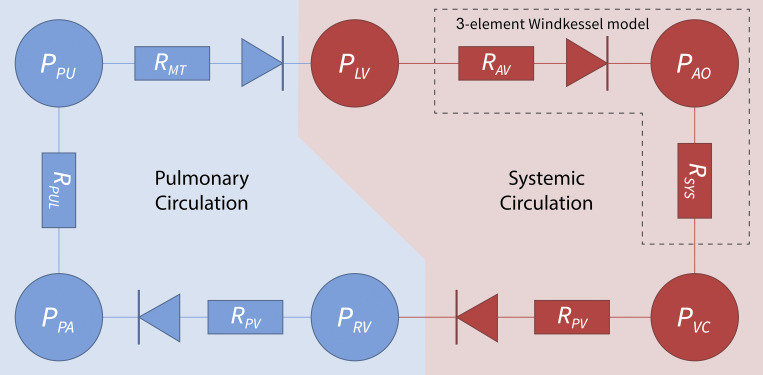
A six-compartment model of the cardiovascular system. The systemic circulation (red) comprises three compartments representing the pressure in the left ventricle (*P*_LV_), aorta (*P*_AO_), and vena cava (*P*_VC_) and three resistors (*R*) representing the aortic valve (*R*_AV_), systemic resistance (*R*_SYS_), and the tricuspid valve (*R*_TC_). The pulmonary circulation (blue) also comprises three compartments representing the pressure in the right ventricle (RV), the pulmonary artery (*P*_PA_), and pulmonary veins (*P*_PU_), and includes the pulmonary valve (*R*_PV_), pulmonary resistance (*R*_MT_), and mitral valve resistance (*R*_MT_). Highlighted is the three-element Windkessel model that considers only the afterload.

The systemic-pulmonary hemodynamic framework (Fig. 1) used in this study to model the cardiovascular system (CVs) consists of two active compartments: the left ventricle (LV) and right ventricle (RV), and four passive compartments: aorta (AO), vena cava (VC), pulmonary arterial (PA), and pulmonary veins (PU).

Each compartment is described by its characteristic elastance, which relates instantaneous pressure and volume (10–12).

Between each compartment are resistors, which represent the resistance to flow of the connecting arterial segments and the inlet and outlet valves of the ventricle (Fig. 1). The systemic portion includes aortic valve resistance (*R*_AV_), systemic resistance (*R*_SYS_), and tricuspid valve resistance (*R*_TC_), and the pulmonary portion consists of pulmonary valve resistance (*R*_PV_), pulmonary resistance (*R*_PUL_), and mitral valve resistance (*R*_MT_). The ventricular inlet and outlet valves are modeled by a diode with zero forward pressure loss to ensure one-way blood flow. For simplicity, neither interaction via the septum between the two ventricles nor arterial inertia was included. The properties of the atria were also not included, as they make relatively small contributions to the overall flow impedance and pressure distribution within the enclosed system (13).

The pressure in the left ventricle was approximated using the law of Laplace (14, 15)

(*1*) $$P_{\text{LV}}=\frac{2hF}{rA},$$

where *F* is the force generated by the muscle (trabecula) ex vivo, *A* is the cross-sectional area of the trabecula, *h* represents an assumed thickness of the ventricle wall, and *r* is the assumed radius of the ventricle. Right ventricular pressure (*P*_RV_) was approximated by multiplying *P*_LV_ by the ratio of the RV-LV end-systolic elastance (Table 1). For each passive compartment, the relationship between instantaneous pressure (*P*) and volume (*V*) was related by its elastance (*E*)

(*2*) $$P_{x}=E_{x}V_{x}\;,$$

where the subscript *x* represents a passive elastic compartment (i.e., AO, VC, PA, or PU). Flow *Q* between compartments was determined by

(*3*) $$Q=\frac{\;P_{\text{up}}-P_{\text{down}}}{R},$$

where *Q* is the flow rate of blood through the artery, *R* is the resistance, *P*_up_ is the pressure in the upstream compartment, and *P*_down_ is the pressure in the downstream compartment. The change of volume of each compartment (including the ventricles) was determined from the flow rates in the arterial sections on either side

(*4*) $$\frac{\text{d}V_{x}}{\text{d}t}\;=\;Q_{\text{in}}-Q_{\text{out}},$$

where d*V*/d*t* is the rate of change of volume with time and *Q*_in_ and *Q*_out_ are the flow rates of blood into and out of the compartment, respectively. To model the ventricular valves, the flow through the valve was constrained to zero if the ventricular compartments and *Q*_in_ and *Q*_out_ were constrained to zero if *P*_down_ exceeded *P*_up_.

**Table 1.** Parameters of the six-compartment cardiovascular system model

Parameter	Symbol	Value	Unit
Elastance end-systolic			
Left ventricular	*E*_ESLV_	170 m	Pa·m^−3^
Right ventricular	*E*_ESRV_	40 m	Pa·m^−3^
Resistance			
Aortic valve	*R*_AV_	5 G	Pa·s·m^−3^
Systemic	*R*_SYS_	175 G	Pa·s·m^−3^
Tricuspid valve	*R*_TC_	2.5 G	Pa·s·m^−3^
Pulmonary valve	*R*_PV_	3.38 G	Pa·s·m^−3^
Pulmonary	*R*_PUL_	12.5 G	Pa·s·m^−3^
Mitral valve	*R*_MT_	2.5 G	Pa·s·m^−3^
Compliance			
Aortic	*C*_AO_	32 p	m^−3^·Pa
Vena cava	*C*_VC_	1.7 n	m^−3^·Pa
Pulmonary artery	*C*_PA_	0.32 n	m^−3^·Pa
Pulmonary venous	*C*_PU_	0.19 n	m^−3^·Pa
Volume			
Total	*V*_t_	24	mL
Stressed	*V*_S_	3	mL
Unstressed	*V*_u_	21	mL
Initial left-ventricular	*V*_LV_	0.23	mL

The total volume of the six-compartment system was divided into stressed (*V*_S_) and unstressed (*V*_u_) volumes. *V*_u_ is the volume each compartment could contain without raising the pressure above zero (11). *V*_S_ is the total volume in each compartment that exceeds *V*_u_.

### Implementing the Systemic-Pulmonary Model

The systemic-pulmonary model was implemented in real-time software architecture to determine the change in muscle length during both the shortening (systolic) phase and the restretching (diastolic) phase of the work-loop. During systole, the shortening phase is comparable to that induced by a Windkessel load (7): when ventricular pressure overcomes that in the aorta, shortening commences and the time course and extent of shortening are determined by the force production of the muscle and the pressure differential of the model. During diastole, the muscle relaxes and when ventricular pressure in the model drops below that of the pulmonary veins, the mitral valve opens, and restretching occurs. The time course of this restretching is determined by the flow rate into the ventricle (i.e., the pressure difference) and the feedback of muscle passive force as the muscle restretches, i.e., the end-diastolic stress-length relation (EDSLR). For in silico validation (appendix) in the absence of a muscle, there was no need to model the EDSLR.

**Figure 2.**
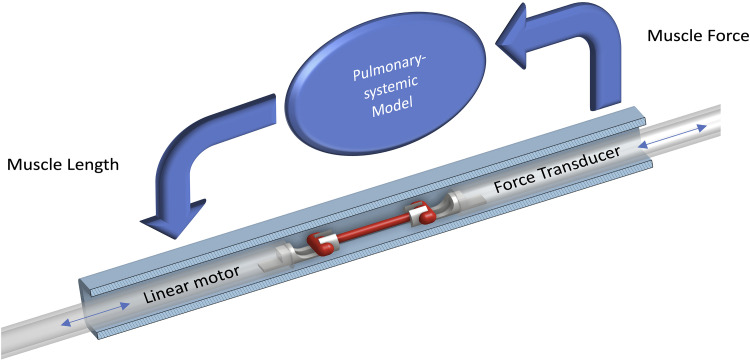
Use of a systemic-pulmonary model to impede trabeculae in our work-loop calorimeter. The muscle (a left ventricular trabecula) was mounted onto hooks, which connect one end of the muscle to a linear motor and the other end to a force transducer. The muscle was positioned in the center of a flow-through bath (shown in section view, square chamber with inner diameter 1 mm) in a calorimeter and stimulated to produce twitch force, which acted as the input to the systemic-pulmonary model (detailed in Fig. 1). The model output determined the position of the linear motor and, therefore, muscle length in real time. Details of the design and functional architecture of this instrument can be found in Taberner et al. (1).

The systemic-pulmonary model was encoded in a real-time control architecture (Fig. 2) within an experimental device (work-loop calorimeter). A detailed description of the design and architecture of our work-loop calorimeter has been published previously (1). The pressures and volumes of, and flow rates between, each compartment were computed in real time at 20 kHz. The twitch force produced by the muscle in the work-loop calorimeter served as the input to the systemic-pulmonary model, converted using Laplace’s law:

(*5*) $$P=\frac{2h\sigma }{r},$$

where *h* is the thickness of the ventricle wall and *r* is the radius of the ventricle. The resulting volume changes from systolic ejection and diastolic filling of the left ventricle in the model were then converted to ventricular radius (*Eq. 6*), which is proportional to muscle length (*Eq. 7*):

(*6*) $$V=\frac{4}{3}\pi r^{3},$$

(*7*) $$L=\frac{r}{r_{\text{o}}}L_{\text{o}}.$$

The maximum muscle length produced by the systemic-pulmonary model was limited to optimal length (*L*_o_) to avoid overstretching the muscle. This output was used to control the linear motor position and, therefore, muscle length. A more detailed description of model implementation in the computational architecture has been outlined previously (7).

### Windkessel-Afterload Model

The performance of the systemic-pulmonary model was compared with that of a three-element Windkessel model of arterial afterload (highlighted portion in Fig. 1). This three-element model (7) considered only the left ventricle and aortic compartments (*P*_LV_ and *P*_AO_) and the atrial valve and pulmonary resistances (*R*_AV_ and *R*_MT_), along with a parameter representing arterial compliance (*C*). In this model, end-diastolic length and restretch rate were constant and defined by the experimentalist, rather than modeled as in the systemic-pulmonary model.

### Parameter Selection

Model parameters (Table 1) were adapted from simulations of the human heart (11) and scaled to represent the rat cardiovascular system based on the systemic Windkessel parameters used in a previous study (7). The scaled parameter set was validated in silico (see Supplementary Material; all Supplemental material is available at https://doi.org/10.17608/k6.auckland.24116526). Model parameters *R*_AV_, *C*_AO_, and *R*_SYS_ are equivalent to the systemic Windkessel parameters *Z*_c_, *C*, and *R*_p_, respectively.

### Experimental Protocol

Animal handling and euthanasia were performed in accordance with the protocol approved by The University of Auckland’s Animal Ethics committee (Ref. No. 002006).

In this methodology study, we conducted proof of concept experiments using a left ventricular trabecula isolated from a male rat heart. To demonstrate reproducibility, we further studied two trabeculae isolated from two other male rats (Wistar strain, 10 wk old, 250–300 g). We thus precluded confounding effects potentially arising from body size, age, or from sex.

Each rat was anesthetized using isoflurane (5% in O_2_), injected with heparin (1,000 IU·kg^−1^), and euthanized via cervical dislocation. The heart was excised and immediately plunged into a chilled Tyrode solution before the aorta was cannulated, and the vasculature Langendorff perfused with Tyrode’s solution at room temperature. The modified perfusate contained (in mM) 130 NaCl, 6 KCl, 1 MgCl_2_, 0.5 NaH_2_PO_4_, 10 HEPES, 10 glucose, 0.3 CaCl_2_, and 20 2,3-butanedione monoxime (BDM). The pH was adjusted to 7.4 using Tris.

The heart was opened along the septum, and trabeculae were dissected from the left ventricle. An appropriately sized trabecula was mounted onto hooks in the work-loop calorimeter device and moved to the measurement chamber. Superfusate containing Tyrode solution with 1.5 mM CaCl_2_ and no BDM was supplied at a constant flow rate of 0.55 µL·s^−1^. Contraction of the trabecula was induced via electrical field stimulation at a rate of 2 Hz. Force was inferred by measuring the deflection of a steel cantilever using laser interferometry and muscle heat output was measured by two thermopile sensors (1, 16). When muscle force had reached a steady state, the muscle was stretched gradually to reach its optimal length (*L*_o_) to achieve maximal active force production.

Muscle dimensions, including *L*_o_ and the major and minor diameters, were measured using a microscope graticule. The three trabeculae, on average, had diameters of 392 ± 40 µm (means ± SD) and perpendicular diameters of 376 ± 10 µm, and *L*_o_ of 3.56 ± 0.14 mm, giving an average cross-sectional area of 0.115 ± 0.012 mm^2^ and an average volume of 0.413 ± 0.053 mm^3^. These dimensions were used to inform the Windkessel model (*Eq. 1*) and to normalize force to stress during postexperimental data processing.

The work-loop calorimeter was enclosed in an insulated chamber to minimize thermal fluctuations and optical disturbances. The ambient temperature within the enclosure was maintained at 32°C using a proportional-integral-derivative temperature controller. This combination of the flow rate of the superfusate, stimulus frequency, and temperature ensured sufficient muscle oxygenation (17).

Each muscle was presented with a mechanical impedance computed from the systemic-pulmonary model. The muscle was initially held under isometric conditions, then supplied with systemic-pulmonary modeled loads from high (800 GPa·s·m^−3^) to low (50 GPa·s·m^−3^), with isometric contractions between each load. The muscle was then presented with the conventional model at user-selected, constant, end-diastolic lengths of *L*_o_ and 0.975 *L*_o_ for a range of *R*_P_ values (800 to 50 GPa·s·m^−3^). The same range of *R*_SYS_ (*R*_P_) values was used for both the systemic-pulmonary and conventional models. Isometric stress-length and heat-stress relations were also measured by reducing muscle length in steps.

After completing both the series of conventional model and systemic-pulmonary work-loop contractions, the muscle was rendered quiescent by halting stimulation. Experimental interventions that involved a series of cyclical muscle length change were performed, which was necessary to correct for the length- and velocity-dependent change in basal heat when determining active heat output (18, 19). Finally, the heat artifact arising from electrical stimulation was determined by measuring the stimulation heat signal in the absence of the muscle in the calorimeter.

### Data Processing

Force was converted to stress (kPa) by division by the mean muscle cross-sectional area of the trabecula. Muscle length was normalized to optimal length (*L*/*L*_o_). Work output was computed as the integral of stress with respect to length throughout a twitch, i.e., the area of the stress-length work-loop. The width of the work-loop gave the extent of muscle shortening. To calculate muscle heat rate (per twitch), thermopile voltage was divided by the product of the thermopile sensitivity (4,000 V/W) and the stimulus frequency (2 Hz). Muscle active heat rate was calculated by subtracting the basal heat rate (arising from the rate and extent of muscle length change) and the electrical stimulation heat rate artifact. Muscle mechanical efficiency was calculated as the ratio of work to the sum of work and active heat, where the denominator was the change of enthalpy.

### Statistical Analyses

Data were plotted as functions of either the relative end-systolic stress (normalized with reference to isometric maxima) or muscle length (relative to optimal length). Data points were fitted using polynomial regression.

## RESULTS

**Figure 3.**
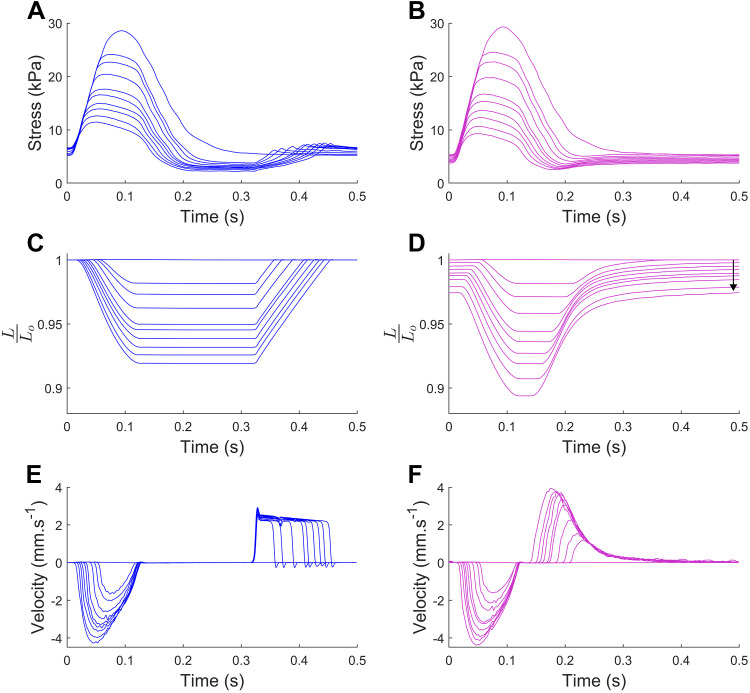
Steady-state stress (*A* and *B*), length (*C* and *D*), and velocity (*E* and *F*) profiles of *n* = 1 left ventricular trabecula contracting against a model-based systemic impedance afterload (*R*_SYS_ = 30 to 800 GPa·s·m^−3^) with constant end-diastolic length (*left*, blue traces) and preload restretching determined by the systemic-pulmonary model (*right*, magenta traces). Arrow indicates the direction of shifting end-diastolic length with decreasing systemic load. The muscle was 3.76 mm in length and had a diameter of 0.38 mm.

Figure 3 shows exemplary data for the stress, length, and velocity profiles for a left ventricular trabecula contracting against a range of systemic resistance afterloads (30–800 GPa·s·m^−3^), for constant end-diastolic length (left, blue traces), and for systemic-pulmonary modeled preload (right, magenta traces). The length traces in Fig. 3*D* illustrate the divergence of the end-diastolic length away from *L*_o_ with decreasing afterload (indicated by the arrow), resulting from the shift in the end-diastolic length set point determined by the model. In comparison, the constant end-diastolic length in Fig. 3*C* results in restretching to the same fixed-end systolic length set point, in this case, *L*_o_, for each afterload. The difference between the Windkessel-afterload model and the systemic-pulmonary model is exemplified in the morphology of the restretch velocity profile (Fig. 3, *E* vs. *F*).

**Figure 4.**
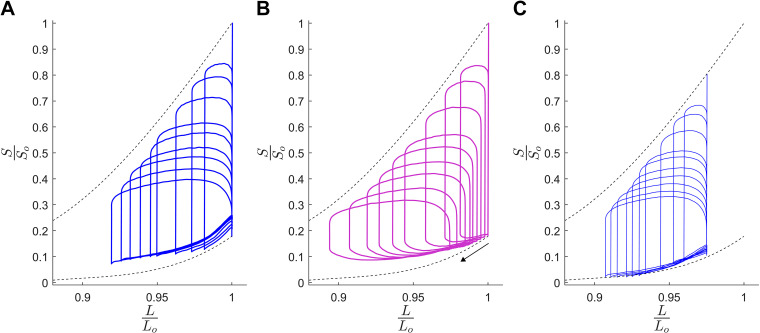
Parametric work-loops for *n* = 1 muscle over a range of systemic afterloads (*R*_SYS_ = 30 to 800 GPa·s·m^−3^) with constant end-diastolic lengths set at optimal length (*L*_o_; *A*) and 0.975 *L*_o_ (*C*), in comparison with the systemic-pulmonary model of variable preloads in *B*. Each loop is a single contraction at steady state. Black arrow in *B* indicates direction of the shift in end-diastolic length as systemic load is decreased. Stress has been normalized to peak isometric stress at *L*_o_, and length normalized to *L*_o_. Black dotted lines denote isometric total stress-length relation (*top*) and isometric passive stress-length relation (*bottom*), as obtained by fitting quadratic regression lines to the data points recorded by reducing muscle length, in 4 to 5 steps, below *L*_o_.

A parametric plot of stress versus length (Fig. 3, *A*–*D*) yields work-loops. The stress-length work-loops from loading with constant preload and the systemic-pulmonary model are displayed in Fig. 4, with the corresponding constant-restretch loops at 0.975 *L*_o_ for the same muscle in Fig. 4*C*. The same values of systemic resistance were used for all three groups of work-loops. Figure 4*B* shows that the reduction of systemic resistance from high resistance (800 GPa·s·m^−3^) to low resistance (30 GPa·s·m^−3^) reduced the preload within the systemic-pulmonary model, corresponding to a reduction of the end-diastolic length (as indicated by the arrow).

### Work-Loop Energetics

**Figure 5.**
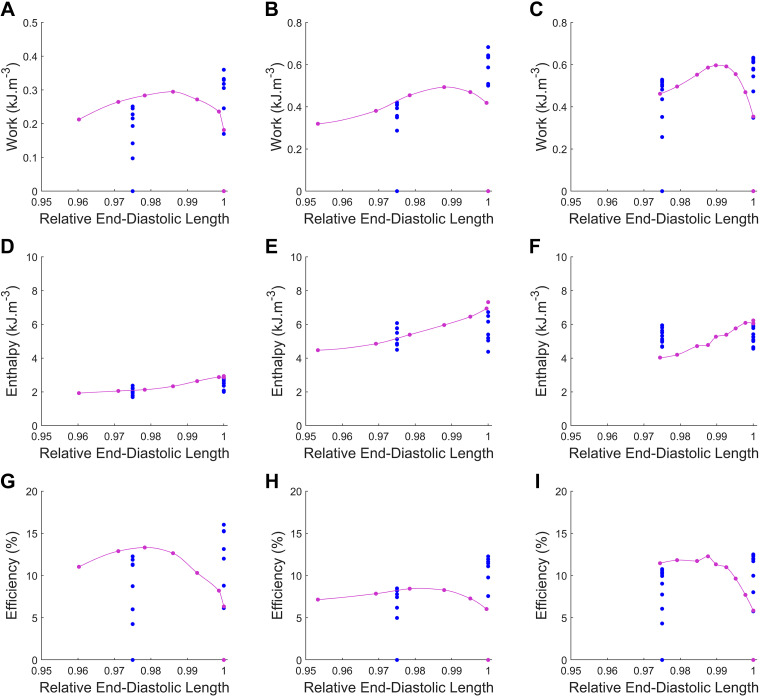
Work (*A*–*C*), enthalpy (*D*–*F*), and mechanical efficiency (*G*–*I*) as function of end-diastolic length for *n* = 3 muscles: *muscle 1* (*A*, *D*, and *G*), *muscle 2* (*B*, *E*, and *H*), and *muscle 3* (*C*, *F*, and *I*). Data are shown for work-loops performed under model-informed (magenta) and fixed-preload [optimal length (*L*_o_) and 0.975 *L*_o_, blue] loading conditions as peripheral resistance (*R*_p_) was varied from 30 to 800 GPa·s·m^−3^.

The area of each work-loop in Fig. 4 is the work done by the muscle during each contraction cycle. By combining this measure with measurements of heat output, the total change in enthalpy of the muscle under each intervention was calculated, along with mechanical efficiency (the ratio of work to total enthalpy). When considering the work, enthalpy, and mechanical efficiency of each muscle with respect to the end-diastolic length, Fig. 5 illustrates the restrictive nature of the user-defined, constant end-diastolic length model (blue) compared with the systemic-pulmonary model (magenta). This highlights the various end-diastolic lengths achieved by modeling both preload and afterload using a systemic-pulmonary model.

**Figure 6.**
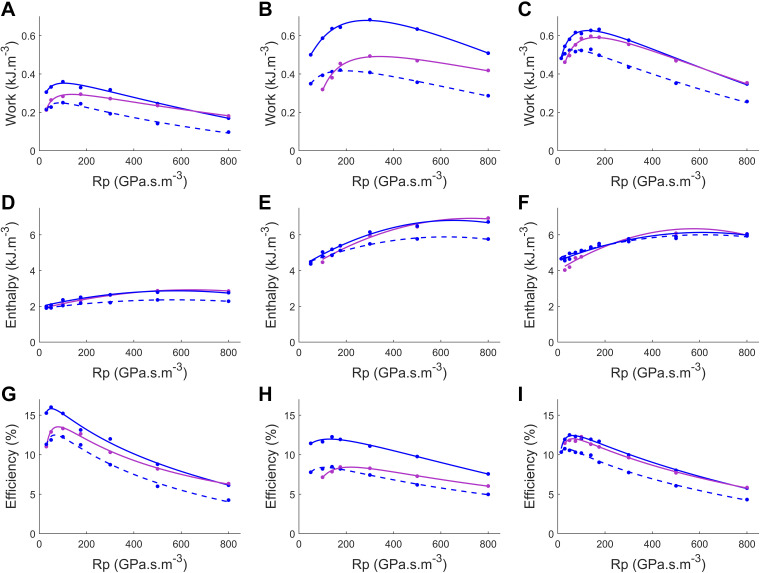
Work (*A*–*C*), enthalpy (*D*–*F*), and mechanical efficiency (*G*–*I*) as function of systemic peripheral resistance (*R*_p_) for *n* = 3 muscles: *muscle 1* (*A*, *D*, and *G*), *muscle 2* (*B*, *E*, and *H*), and *muscle 3* (*C*, *F*, and *I*). Work-loops were performed using a systemic-pulmonary model-based load (magenta) or with constant end-diastolic length (blue), restretched to optimal length (*L*_o_; solid) and 0.975 *L*_o_ (dashed), as *R*_p_ was varied from 30 to 800 GPa·s·m^−3^. Work and efficiency data were fitted by cubic regression with the log of the systemic resistance and enthalpy data by quadratic regression.

**Figure 7.**
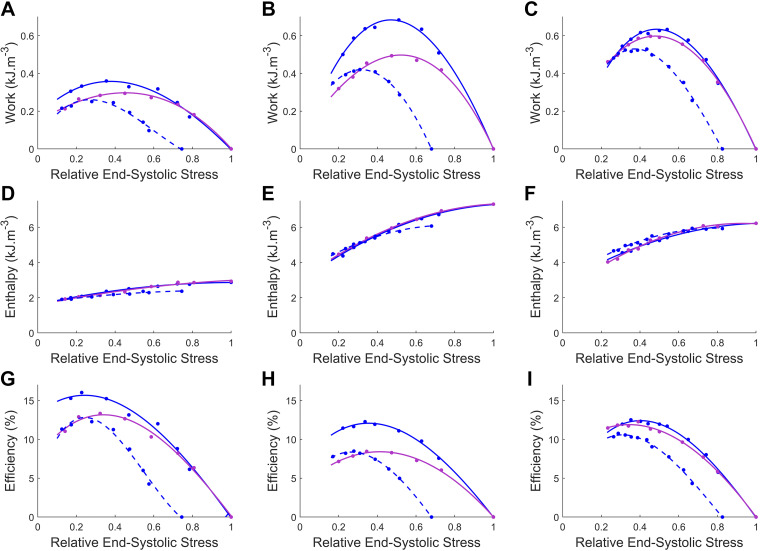
Work (*A*–*C*), enthalpy (*D*–*F*), and mechanical efficiency (*G*–*I*) as function of end-systolic stress (relative to optimal stress obtained under the isometric contraction) for *n* = 3 muscles: *muscle 1* (*A*, *D*, and *G*), *muscle 2* (*B*, *E*, and *H*), and *muscle 3* (*C*, *F*, and *I*). Work-loops were performed using a systemic-pulmonary model-based load (magenta) or with constant end-diastolic length (blue), restretched to optimal length (*L*_o_; solid), and 0.975 *L*_o_ (dashed), as peripheral resistance (*R*_p_) was varied from 30 to 800 GPa·s·m^−3^. Work and efficiency data are each fitted with cubic regression, and enthalpy data were fitted with quadratic regression.

Mechanoenergetics measured from the three muscles employed in this study were plotted against systemic resistance (*R*_p_, Fig. 6) and relative end-systolic stress (Fig. 7). In both figures, the shift of the end-diastolic length arising from the systemic-pulmonary model resulted in work and efficiency relations that bridged the region between the static preloads. This observation is exemplified by the work and efficiency curves aligning with the *L*_o_ curves for high loads, shifting to coincide with the 0.975 *L*_o_ curve as the end-diastolic length decreases with decreasing load. This implies that the variable end-diastolic length resulted in neither peak work nor efficiency values as high as those of the loops performed at *L*_o_ nor as low as those at 0.975 *L*_o_. Instead, it resulted in curves with broader “optimal” ranges of work and efficiency values. There is no apparent difference in the change of enthalpy between the constant end-diastolic length model at optimal length and the systemic-pulmonary model (Figs. 6 and 7, *D–F*); however, the mechanical efficiency differs (Figs. 6 and 7, *G–I*). These results indicate that work output has the greatest influence on muscle mechanical efficiency.

## DISCUSSION

In this study, we present a systemic-pulmonary model of the cardiovascular system, computed in real time, for the application of loading isolated trabeculae in real time to perform force-length work-loops. The model was parameterized by performing in silico simulations (Supplemental Figs. S1 and S2). These simulations validated the formulation of the systemic-pulmonary model for the application of rat model parameters. This model was deployed in our work-loop calorimeter control system (Fig. 2) and allows, for the first time, measurement of work-loops from isolated cardiac muscle in which both the ejection and refilling phases are determined by real-time model-based loads (Figs. 6 and 7). We tested the systemic-pulmonary hemodynamic model by conducting in vitro muscle experiments where muscle mechanics and energetics, specifically active heat rate, were measured to enable the calculation of muscle mechanical efficiency. We assessed muscle mechanoenergetics outputs by comparing the loading protocols between using the systemic-pulmonary model and an existing systemic Windkessel model that accounts only the shortening phase of work-loop (7, 20). Several notable observations are presented when muscle mechanoenergetics outputs are evaluated in ranges of peripheral resistance (Fig. 6), end-systolic stress (Fig. 7), and, more markedly, end-diastolic length (Fig. 5).

### Model Considerations

The four considerations of this approach include the dynamics of ventricular refilling during diastole, coupling between afterload and preload, instantaneous PV behavior of both ventricles, and computation time. To model the dynamics of ventricular filling during diastole, we have acknowledged that the passive mechanics of the muscle contribute to the end-diastolic PV relation (see methods). Both the time course of ventricular refilling and the extent of refilling are determined by preload (21), which arises from the pressure exerted by the venous return to each ventricle and, therefore, setting the extent of refilling achieved before the next contraction (22). In vivo, preload is dynamic and depends on various factors, predominantly ventricular compliance, heart rate, sympathetic nervous response, and, more importantly, preload is coupled with afterload (21, 23). In ex vivo whole heart experiments, however, preload is usually fixed or set by experimentalists and held to be independent of afterload (24–26). The same limitation is likewise executed in experiments studying isolated papillary muscles (2) and trabeculae (8, 27, 28), where the preload is usually set fixed at the optimal length of the muscle. In our systemic-pulmonary model, we consider the influence of both the preload and afterload forces on the formation of a work-loop, determining the dynamics of both the shortening and restretching phases. Furthermore, we assess the implications of coupling between the preload and afterload on the mechanics and energetics of work-loop contraction in vitro and the effect of model-based variations in preload and afterload occurring simultaneously, as demonstrated in Fig. 4.

In considering the cardiovascular system as a closed-loop system, we have acknowledged that variations in the impedance of one compartment (e.g., by varying systemic arterial impedance) affect the distribution of fluid throughout the whole system. Therefore, linking the outflow from the left ventricle back to the left ventricular inflow via the right ventricle and systemic/pulmonary circulatory systems is prudent. Such an afterload-preload coupling is achieved in the model using a lumped-parameter representation of the entire circulatory system. This includes the left and right ventricles as active compartments whose pressure dynamics are informed by the muscle force twitch and four passive elastic compartments representing the primary volumes stores of the circulatory system.

Central to the model is the description of ventricular function consistent with the time-varying elastance theory (29, 30) to modulate volumes and pressures in the ventricles based on their PV relations and a normalized activation function. The instantaneous PV behavior of each ventricle under any loading conditions can be simulated using an activation function and the ventricle-specific end-systolic PV relations (12). Aside from the active ventricular compartments, four passive compartments represent the arteries and veins in the systemic and pulmonary circulations, each having a PV relation determined by their respective elastance. The pressure gradient and the resistance of the connecting arterial portions mediate the flow between compartments, with the restriction of only forward flow allowed through the four ventricular valves of the heart.

Regarding computation time, a simple lumped-parameter model that determines hemodynamic pressures and flow rates in a system is advantageous when striving to compute real-time solutions (13). Six-compartment PV models of the cardiovascular system comprise a series of Windkessel-type units. The associated parameters are compartment specific but provide a lumped-parameter estimate of pressure and volume distributions throughout the system when computed in conjunction. Various six-compartment models (11–13, 31–33) effectively constitute a collection of connected Windkessel models, which synchronously determine the distribution of blood in different compartments. These mathematical models form the basis for the method adopted here. However, we advanced this technique by computing in real time, which was made possible by encoding the model in a real-time software and hardware-based operating system (LabVIEW, National Instruments). This advancement to real-time computing allowed for the application of outputs from the model to in vitro experimental preparations, enabling the first even application of a loading system that couples shortening and restretching of the muscle in this manner.

### Applications of the Model

The systemic-pulmonary model of the cardiovascular system provides a valuable tool for assessing isolated cardiac muscle function. The novel real-time loading method allows simultaneous modulation of end-diastolic length (modulated by preload) and end-systolic stress-length behavior in response to a change in systemic peripheral resistance (afterload). Varying end-diastolic length in response to a model-based approximation of pulmonary venous return pressure resulted in work and efficiency curves that traverse a path between the static relations (Fig. 5). This traversal results in a broader range of loads around the peak where work varies minimally with systemic resistance compared with the constant end-diastolic length curves. For example, in Fig. 7*A*, the work output is relatively consistent within the relative end-systolic stress range of 0.2–0.6 before dropping off at either side. For the constant end-diastolic length plots, the plateau in work occurs between 0.2 and 0.5 relative end-systolic stress for data at *L*_o_ and from 0.2 to 0.4 relative end-systolic stress at 0.975 *L*_o_. This broader plateau could reflect an adaptation in matching the ventricle and arterial impedance load to supply a semicontinuous work output despite the continuous load fluctuations. If so, a more extensive working range of loads that result in minor changes in work output and energy expenditure would be advantageous in weathering the constant variations of cardiovascular load in vivo (34).

Our systemic-pulmonary model begins to restretch the muscle before the muscle is fully relaxed, i.e., when the active muscle force is still decreasing (Fig. 4*B*). In contrast, the user-defined restretch in the Windkessel-afterload model maintains end-systolic length until muscle force has relaxed to a minimum, before restretching. The muscle thus performed lower work per twitch against the systemic-pulmonary model. This difference will have some impact on muscle energetics, as enthalpy remains the same but corresponds to a lower work output (particularly the work-loop at *L*_o_), resulting in reduced efficiency. However, at 0.975 *L*_o_ the work and efficiency are higher than that with constant end-diastolic length. The difference in energetics between the restretch modes highlights the preference for the more physiological representation of the diastolic portion of work-loop dynamics achieved by our systemic-pulmonary model, and suggests an opportunity to study the link between the timing of diastolic restretching and energetics.

### Existing Mathematical Models

The present study is the first to apply a systemic-pulmonary model on isolated cardiac muscle. Systemic-pulmonary hemodynamics have been modeled in a mathematical modeling environment (11–13, 32), and our model shares some similarities with these. Burkhoff and Tyberg (11) presented one of the first examples of such a model, describing the volume distribution throughout a human cardiovascular system. This method formed the basis of our approach, in combination with the lumped-parameter real-time representation previously employed for the representation of only arterial afterload, using a three-element Windkessel approach (7).

### Limitations

The complexity of our model could be increased to include inductive terms to represent areas of turbulent blood flow (e.g., at the ventricular valves), such as in the work of Smith et al. (10, 13). Interaction between the left and right ventricles via the septal wall also impacts ventricular pressure and has been successfully included by others in this form of model (13, 31). Furthermore, the spherical geometrical assumption using the law of Laplace could be overcome by leveraging existing mathematical models that better represent ventricular geometry (35). However, the real-time nature of the model implementation places significant limitations on the possibility of increasing complexity. A further potential limitation of this study lies in the derivation of the systemic-pulmonary model parameters. These parameters used in this study were based on that of a 75-kg human male (11) which were converted to reflect that of a male rat, determined by our previous experiments (7, 20).

### Sex Specificity as a Future Application

For this study, we have only included male muscle samples and, therefore, used male model parameter values. However, for future applications, care should be taken to consider sex as a biological variable, and studies planned accordingly. Careful consideration should be given to the effect of sex in muscle sample and in the model parameter selection, including the variations which may exist in the CVs hemodynamic parameters in correlation with the estrus cycle.

When considering sex differences in the muscle sample, there is some evidence that suggests no difference in both the isometric peak active and passive force production, nor the rates of rise and fall of contractile twitch, in isolated rat muscles over the entire physiological working range of muscle length (36), and at the adult age (<4 mo) (37, 38). Contrariwise, there is also evidence that some contraction characteristics, namely, the magnitude of force production and of calcium transient, can be modulated by estrus stage (39). Our systemic-pulmonary model could potentially be used to reconcile this inconsistency when model parameters are adapted appropriately where, for example, arterial load (40) and aortic stiffness, *Z*_c_ (41), are both greater in women.

When considering model parameters, it has been shown that vascular function, including but not limited to basal vascular tone and vascular compliance, are not only sex dependent but also strongly age dependent in females. Incidences of cardiovascular disease (hypertension and stroke) are lower in premenopausal females, yet increase in post menopause to surpass both premenopausal females and age-matched males (42). This could be linked to the effects of sex hormones on regulation of the vascular network (40, 41) and would therefore influence the parameters that should be used in this systemic-pulmonary hemodynamic model. A future application of this experimental method would allow the investigation into the implications of sex hormones on the mechanics and energetics of work-loops when coupled with model parameters that include not only sex specificity but also estrus specificity for female systems.

### Conclusions

A systemic-pulmonary model of the cardiovascular system was constructed to simulate venous return pressure, or preload, to provide a model-based estimate of cardiac muscle end-diastolic length. This variable preload length couples the preload and afterload experienced by the left ventricle in vivo, enabling the application of a closed-loop loading system for isolated cardiac muscle experiments. The implication of model-based variable end-diastolic length on work and efficiency outputs indicates a potential adaptation of the ventricle as a pump source to operate over a range of loads while achieving comparable work and efficiency outputs. A possible application of the model includes examining the hemodynamic coupling between arteries and ventricular musculature considering systemic-pulmonary interactions in an in vitro setting.

## APPENDIX

### In Silico Parameter Validation

Simulations were performed to test the performance of the systemic-pulmonary model and to validate the parameters used to mimic rat cardiac physiology. The in silico model was implemented in MATLAB (MathWorks).

The in silico model architecture was similar to that described in Fig. 1, with some necessary changes made to the active compartments. In the in silico model, as there was no physical muscle to generate a force twitch, the pressure (*P*) developed in each ventricle was instead modeled by *Eq. A1* using the time-varying elastance theory (29, 30)

(*A1*) $$P=e\left(t\right)E_{\text{ES}}(V-V_{0}),$$

where *e*(*t*) is the activation function (a normalized isometric force twitch measured from an isolated rat trabecula; Supplemental Fig. S1), *V* is the current ventricle volume, *V*_0_ is the volume of the ventricle when ventricular pressure is zero, and *E*_ES_ is the ventricle-specific end-systolic elastance, defined as the slope of the end-systolic pressure-volume relation. This relation is usually approximated to be linear within low volume ranges (43, 44), although experimentally, it is curvilinear over greater volume ranges (45, 46). For these simulations, the equation was simplified by assuming *V*_0_ to be zero, which constrained the end-systolic relation to intersect the origin of the pressure-volume (PV) axis.

The use of a mathematically prescribed ventricular PV relation during in vitro experiments was unnecessary since the trabecula determined the stress-length relation. Hence, the simplified end-systolic PV relation was only used for model development and simulation purposes. There was also no need to explicitly model the end-diastolic PV relation because the passive behavior is inherent to the isolated muscle sample during in vitro experiments.

For the simulation, the force twitch was modeled at a rate of 2 Hz (Supplemental Fig. S1). The in silico model was run until the volume distribution between compartments reached a steady state. Supplemental Fig. S2 illustrates an example of the simulation output, adopting the parameters (Table 1) for pressure and flow rates relevant for determining the dynamics of left ventricular PV loops. Aortic pressure (*P*_AO_) represents afterload, defined as the pressure immediately downstream from the left ventricle that must be overcome by the left ventricle to initiate ejection. The rate and dynamics of this ejection (*Q*_AV_) were therefore determined by the pressure gradient formed across the aortic valve between *P*_LV_ and *P*_AO_. The venous return pressure determined the preload via the flow rate through the mitral valve (*Q*_MT_), which arose from the pressure difference between pulmonary venous pressure (*P*_PU_) and *P*_LV_ during diastole. Therefore, this venous return pressure determined the end-diastolic volume before the next contraction.

Supplemental Fig. S3*A* displays simulated PV loops for *R*_p_ = 175 GPa·s·m^−3^ (solid) and *R*_p_ = 115 GPa·s·m^−3^ (dashed line), produced by the systemic-pulmonary model using the parameters in Table 1. Each loop exhibits a different initial volume and afterload. Also shown are loops for a range of *R*_SYS_ values, with the arrow indicating an increase of systemic resistance from low values (50 GPa·s·m^−3^) to high values (500 GPa·s·m^−3^). The resultant loops exhibit simultaneous increases in both preload and afterload, as would be expected and have been observed previously experimentally (29, 47).

These in silico results provided confidence in the derived parameters (Table 1) and enabled us to commence in vitro experiments on isolated trabeculae.

## DATA AVAILABILITY

The experimental data contributing to this study can be made available by the corresponding author upon request.

## SUPPLEMENTAL DATA

10.17608/k6.auckland.24116526 Supplemental Figs. S1–S3: https://doi.org/10.17608/k6.auckland.24116526.

## GRANTS

The work was supported by University of Auckland Doctoral Scholarships (to A.S.G. and J.D.), Royal Society of New Zealand Marsden Fast-Start Grant UOA1504 and Marsden Project Grant MFP-UOA2206 (to J-C.H.), Health Research Council of New Zealand Sir Charles Hercus Health Research Fellowship Grant 20/011 and Explorer Grant 21/758 (to J-C.H.), and Royal Society of New Zealand James Cook Research Fellowship (to A.J.T.).

## DISCLOSURES

No conflicts of interest, financial or otherwise, are declared by the authors.

## AUTHOR CONTRIBUTIONS

A.S.G., J.D., A.J.T., and J-C.H. conceived and designed research; A.S.G. performed experiments; A.S.G. analyzed data; A.S.G. interpreted results of experiments; A.S.G. prepared figures; A.S.G. drafted manuscript; A.S.G., J.D., A.J.T., and J-C.H. edited and revised manuscript; A.S.G., J.D., A.J.T., and J-C.H. approved final version of manuscript.
